# Understanding the psychosocial burden of living with advanced COPD in context of palliative care: A mixed methods study

**DOI:** 10.1177/13591053251316504

**Published:** 2025-03-13

**Authors:** Barbara Gonçalves, Joanne Lusher, Audrey Cund, Caroline Sime, Eileen Harkess-Murphy

**Affiliations:** 1 University of the West of Scotland, UK; 2 Regent’s University London, UK; 3 Scottish Partnership for Palliative Care, UK

**Keywords:** biopsychosocial model, COPD, mixed methods, palliative care, psychological distress, quality of life

## Abstract

People with advanced chronic obstructive pulmonary disease (COPD) face substantial physical and psychosocial challenges influencing their quality of life. This study explored the psychosocial aspects of people with COPD attending palliative care services. Concurrent embedded mixed methods study with 22 individuals was conducted. Data collection involved semi-structured interviews and McGill Quality of Life-Revised and Hospital Anxiety and Depression Scale questionnaires. Findings revealed a negative correlation between quality of life and psychological distress, emphasising that as disease severity increased, so did psychological distress. From interviews emerged three themes: adjustment to living with a debilitating disease, loss of identity and developing lifestyle coping strategies. To conclude, a biopsychosocial perspective to understanding the impact of COPD is essential in identifying mitigating factors which exacerbate disease burden and increase psychological distress of people living with COPD. Implementing a biopsychosocial framework can enhance care by furthering self-management, reducing psychological distress and promoting a person-centred approach.

## Introduction

Chronic obstructive pulmonary disease (COPD), is a chronic inflammatory respiratory condition and one of the leading causes of death, imposing a significant burden worldwide (Adeloye et al., 2022; Iheanacho et al., 2020). Individuals with COPD exhibit a spectrum of respiratory diseases, including clinical chronic bronchitis and pathological emphysema, often accompanied by additional extra-pulmonary comorbidities substantially affecting quality of life (QOL) of patients (Brien et al., 2018; Giacomini et al., 2012). People with COPD face daily challenges due to physical limitations, resulting in reduced independence, increased reliance on the assistance of others and assistive devices and emotional distress (Cannon et al., 2016; Ek and Ternestedt, 2008).

The biopsychosocial model of health and illness, proposed by Engel (1977, 1980), suggests that biological, psychological and social factors play fundamental roles in health and disease and that to understand one’s health comprehensively, it is advisable to assess all these multifaceted aspects. This framework is especially applicable to individuals with chronic diseases like COPD, as it corresponds to all aspects of their lives, which actively intertwine and provides a progressive and holistic illness model (Wade and Halligan, 2017).

The biological level of COPD is affected by numerous physical symptoms which are correlated with decreased QOL, social and psychological distress (Fei et al., 2022; Jain et al., 2020; Weiss et al., 2023). Breathlessness stands out as the defining difficulty for COPD patients, restraining their physical functioning and lifestyle activities due to the sensation of heaviness and increased effort to breathe, significantly affecting their day-to-day activities, health status and QOL (Hanania and O’Donnell, 2019; Johansson et al., 2019). Even slight efforts triggering breathlessness and associated COPD symptoms not only impede social engagement but also evoke distressing anxiety, leading to limitations in daily activities due to insecurity about potential future attacks and fears of suffocation and death (Avşar and Kaşikç, 2011; Christiansen et al., 2023; Strang et al., 2014).

In the biopsychosocial framework, a vulnerability model conceptualises the interface between biological and psychosocial factors in chronic illness, in which minor chronic stressors added to extra daily stressors can promote a disease episode (Lu et al., 2012; Ng et al., 2007; Sinn et al., 2016). Within this framework, a ‘dyspnoea-anxiety-dyspnoea cycle’ represents a vicious cycle of breathlessness and anxiety, signifying an emotional reaction to breathlessness, where anxiety, in turn, exacerbates the sensation of breathlessness (Bailey, 2004). Furthermore, Willgoss et al. (2012) revealed a complex and perplexing interaction between anxiety and dyspnoea, leading to deteriorating symptoms and deconditioning, identifying two anxiety types: one due to breathlessness (dyspnoea-related anxiety) and the other causing breathlessness triggered by other factors. Regardless of the trigger, anxiety and panic attacks were linked to intense fear, loss of control and hopelessness, leading to loneliness and social isolation, aligning with the biopsychosocial model. Aggravated anxiety can also be attributed to other psychological causes, such as reliance on others, loss of control, fear of the future and death anxiety, potentially leading to a phobic avoidance of physical activity (Strang et al., 2014; Togluk and Çuhadar, 2021; Yohannes and Alexopoulos, 2014). Having depressive symptoms is related to more profound anxiety symptoms and, in people with COPD, can be characterised as feelings of despair and pessimism, social withdrawal, decreased sleep and appetite, increased apathy, problems with concentration and performing daily life activities, poor functional performance, lower self-reported health and poor self-management of exacerbations (Aldhahi et al., 2023; Aras et al., 2017; Laurin et al., 2012). Subsequently, it may induce patients’ reactions to the losses, including functional effects such as withdrawing from occupational activities, role rearrangement in the family and fear of becoming a burden (Avşar and Kaşikç, 2011; Gysels and Higginson, 2011; Sigurgeirsdottir et al., 2020).

Psychological distress is strongly connected with social networks (Pearce et al., 2023; Santini et al., 2015). Likewise, social isolation and loneliness can be a backwash of depressive symptoms (Elmer and Stadtfeld, 2020; Luo, 2023). Perceived isolation was indicated as an anchor through which social disconnectedness affects mood disorders, and through which depression and anxiety advance social withdrawal (Santini et al., 2020). Similarly, chronic disease negatively changes family interactions and social connections and has consequences in exerting stressful emotions and more negative life events (Zhang et al., 2017). This casts a light on the interconnections of social ties and mood, which may help indicate which strategies might be the most beneficial in improving patients’ mental health and well-being.

This study sought to answer several key questions. Firstly, in the quantitative arm, it explored the factors that correlate with QOL and psychological distress of people living with advanced COPD. Secondly, in the qualitative arm, the study explored the lived experiences of COPD patients attending palliative care. Lastly, the mixed methods triangulation, examined what specific factors affect the quality of life of people living with advanced COPD.

## Methodology

### Design

A concurrent embedded mixed methods one-phase design with a parallel variant was used in this study (Creswell and Plano Clark, 2011; Plano Clark et al., 2013). Two parallel strands were gathered and analysed independently and separately, and were only joined during the interpretation (Edmonds and Kennedy, 2013). Given that the COPD trajectory is progressive, the concurrent design was selected, wherein both types of data were collected in one phase to capture the current state of a patient. This study embraced pragmatism as the optimal paradigm and taking this view meant that the problem studied and the questions asked about them are more important than the method or philosophical worldview that guides the approach. The quantitative arm, a secondary research design, was designed to match the methodological requirements of the qualitative design. Therefore, this study was designed as a quantitative, cross-sectional study with two questionnaires, embedded within a primary qualitative research design, which was a descriptive study with semi-structured interviews. This study adhered to the Mixed Methods Article Reporting Standards (Levitt et al., 2018).

### Participants and procedures

This research was conducted in West and Central Scotland, United Kingdom. Purposive sampling was employed to specifically target individuals with advanced COPD receiving palliative care services at both generalist and specialist levels. Advanced COPD refers to the later stage of the condition when symptoms become progressively worse and no longer respond effectively to treatment (Zhou et al., 2015). Participants were included if they had doctor-diagnosed COPD (stage II–IV), and attended a respiratory clinic or hospice with at least two visits. Patients with moderate, II GOLD stage were recruited if attended the service for several years. Exclusion criteria were psychiatric or cognitive disorders (excluding depression, anxiety and panic attacks), other respiratory diseases, progressive neurological or neuromuscular disorders, active cancer or other uncontrolled diseases.

Potential participants underwent initial screened by an appointed person from the medical team prior to being contacted by them in person or by phone for voluntary participation in the study. They were given a Participant Information Sheet outlining the goals and objectives of the study. Recruitment took place in four services, through one Breathe Easy clinic which held monthly disease-specific support groups, and 3 day hospices services attended by patients with various diseases, both malignant and non-malignant, for a 12-week programme and a pulmonary rehabilitation programme.

### Ethics approval and consent to participate

Ethics approval was obtained from London Stanmore NHS Ethics Committee (reference 19/LO/255972) and all participants signed an informed consent.

### Data collection

The study was conducted in person by the first author, a qualified healthcare professional, impartial from the recruiting services. Data were collected between June and November 2019 at the participants’ homes (*n* = 12), in private rooms within healthcare facilities (*n* = 9) and at their workplace (*n* = 1). Two participants were accompanied by spouses. Questionnaires were followed by interviews which were transcribed verbatim and anonymised, with no interview repetitions. Field notes were also taken and recruitment continued until data saturation was reached.

### Measures

Demographic questionnaires were collected to gain a better understanding of the sample and to establish information such as age, gender, smoking status, marital status, level of education, working status, disease severity (presented with GOLD classification and FEV1% pred.) and current medications.

The McGill Quality of Life Questionnaire-R (MQOL-R) was employed to measure QOL (Cohen et al., 2017). MQOL-R contains 15 items measuring four domains: physical, psychological, existential and social, capturing preceding 48-hour period. The tool starts with a single item assessing overall subjective QOL. The MQOL-R total score is the mean of the four subscale scores. Each subscale score is the mean of the items founding that subscale. All MQOL-R items range from ‘0’ to ‘10’ on a Likert scale, indicating that the lower the score, the lower the reported QOL.

Hospital Anxiety and Depression Scale (HADS) was used to measure depression and anxiety (Zigmond and Snaith, 1983). It is a self-assessment 14-item questionnaire which measures depression and anxiety in non-psychiatric groups. HADS has two 7-item subscales assessing depression and anxiety in the preceding week. Each HAD-D (depression) and HAD-A (anxiety) has a score from a total of 0–21, which can be tallied to give a total anxiety-depression (HADS total) score with a maximum of 42 points. Items are placed on a 4-point Likert scale, with zero denoting not present to 3 representing severe symptoms. Scores of 11 or higher on either subscale indicate a significant ‘case’ of psychological morbidity, while scores of 8–10 are classified as ‘borderline’, and scores of 0–7 are considered ‘normal’ (Zigmond and Snaith, 1983).

Semi-structured interviews were audio-recorded and were conducted using a guide with open-ended questions on participants’ day-to-day experiences, social interactions, relationships, psychological well-being, satisfaction with healthcare services and general patient needs and any extra insights provided by the participants.

### Data analyses

A series of correlations were run in SPSS 25 to determine whether there were any significant relationships between QOL scores (dependent variable) and other variables, such as demographic information and psychological distress (independent variables; Field, 2018). Kendall tau correlation was computed with demographic data and with scores of HADS, its subscales, as well as with the total score and subscales of MQOL-R.

Framework analysis was employed to analyse interviews. Following the procedure of analysis presented by Gale et al. (2013), the first author conducted transcription, familiarisation, open coding, development of a working analytical framework, application and charting of data into this framework and final interpretation to identify emerging themes. Transcripts were coded into themes using NVivo12, followed by the development of a working analytical framework reviewed by co-authors. The framework was applied to index transcripts and data were charted in a matrix. Interpretation involved seeking correlations and mapping codes, with manual tables created to examine associations among concepts. Eight preliminary themes were identified and refined into three distinct code groups, with subsequent subthemes. All processes of the analysis were supervised by co-authors who provided independent scrutiny and feedback to ensure reliability, minimise bias and uphold the validity and rigour of the findings. Throughout, participants did not contribute feedback or corrections to the transcripts or findings.

Methodological triangulation was employed in this study (Bekhet and Zauszniewski, 2012). Following the approach outlined by Creswell and Creswell (2018), the integration was conducted using ‘a side-by-side comparison’ and was presented in a narrative format. Similarities and differences were discussed by comparing one set of results with the other.

### Participants characteristics

The study had 22 participants, equal for both arms of the study. Of the 26 advanced COPD patients approached at the Breathe Easy clinic, six declined, two had lung infections, one was unreachable and one passed away before contact. From the day hospice group, six of 11 eligible patients participated. Twenty-two participants (*n* = 12 female, *n* = 10 male) were involved. Eleven participants were married, five divorced or separated, four widowed and two single. Fourteen had general secondary education, five vocational, two higher and one did not disclose. Eighteen were retired, three unemployed and one employed. Most were ex-smokers (*n* = 18), with three current smokers and one exposed to second-hand smoke. The mean age was 67.23, and FEV1 pred. was 36.92%. Three had mild COPD, ten severe and seven very severe (two missing data). Twelve participants were oxygen-dependent.

## Results

### Quantitative findings

The results of QOL demonstrate that this sample was most affected by physical aspects and least by social factors. Table 1 displays scores for anxiety and depression and quality of life subscales.

**Table 1.** Descriptive statistics of quality of life and psychological distress.

Questionnaire	Score
MQOL-R Total score	5.84 ± 2.17
MQOL-R Physical subscale	4.30 ± 2.28
MQOL-R Psychological subscale	5.48 ± 3.04
MQOL-R Existential subscale	5.93 ± 2.59
MQOL-R Social subscale	7.74 ± 2.39
MQOL-R Self-assessed QOL	4.95 ± 3.04
HADS Total score	18.95 ± 7.88
HAD-D	8.81 ± 4.29
HAD-A	10.14 ± 4.79

Correlation tests were conducted to examine if there was a significant relationship between QOL and anxiety and depression among COPD participants. The findings from correlation analysis indicate that the type of service attended at the time of the study, disease severity, gender, age and oxygen use were not significantly associated with QOL (Table 2). MQOL-R total score was significantly correlated with the HADS total score (*r* = −0.560, *p-*value = 0.000), with its subscales HAD-D (*r* = −0.689, *p*-value = 0.000) and HAD-A (*r* = −0.325, *p*-value = 0.040). HADS total score was used further for examination.

**Table 2.** Correlation of type of service, gender, age, disease severity, oxygen use, HADS with MQOL-R total score.

Variable	Kendall’s tau correlation coefficient	*p*-Value
Type of service	0.283	0.121
Gender	0.114	0.531
Age	0.026	0.865
FEV1% pred.	−0.298	0.069
GOLD classification	0.344	0.062
Oxygen use	−0.157	0.391
Anxiety (HAD-A)	−0.325	0.040
Depression (HAD-D)	−0.689	0.000
Anxiety and depression (HADS)	−0.560	0.000

Correlation relationships of HADS total and type of service, gender, age, oxygen use and severity, revealed that no significant relationship existed between the type of service attended, gender, age, or severity classified with GOLD stages (Table 3). The findings demonstrate that as severity increased, so did HADS total; however, it is a weak association. The severity measured with FEV1% pred. was significantly related to HADS total (*r* = 0.346, *p*-value = 0.037) and GOLD classification showed a negative association (*r* = −0.336, *p-*value = 0.072), suggesting that a more advanced stage tended to be related to a higher HADS total score, although this correlation was not statistically significant.

**Table 3.** Correlation of type of service, gender, age, disease severity and oxygen use with HADS total score.

Variable	Kendall’s tau correlation coefficient	*p*-Value
Type of service	−0.198	0.284
Gender	−0.030	0.869
Age	−0.062	0.691
FEV1% pred.	0.346	0.037
GOLD classification	−0.336	0.072
Oxygen use	−0.152	0.409

### Qualitative findings

The semi-structured interviews averaged 42 minutes in length and ranged between 25 and 105 minutes. The emergent themes are presented in Table 4.

**Table 4.** Themes and subthemes.

Theme	Subthemes
Adjustment to living with a debilitating disease	• Experiencing physical symptoms • Experiencing anxiety, panic attacks and depression • Interrelationship of physical and mental experiences • Changes in lifestyle
Loss of identity	• Loss of social life • Loss of social and family roles • Social isolation and loneliness • Loss of independence
Developing lifestyle coping strategies	• Accepting the disease • Looking for purpose • Trying to maintain a normal lifestyle

#### Adjustment to living with a debilitating disease

Living with COPD had a significant impact on the lives of the participants in the study and led to a change in their lifestyle. Physical symptoms substantially contributed to the increase in their life limitations. All participants experienced symptoms of dyspnoea daily, and its intensity varied from day to day. Shortness of breath reduced the individuals’ mobility and considerably prevented them from doing what they were previously able to do.

The shortness of breath is the biggest thing, the constant shortness of breath. It used to be for a certain length of time, now it’s as soon as I get up and start walking.

Participant 14

Participants experienced various physical symptoms, including coughing, tiredness, joint pain and mobility problems. Many had trouble sleeping due to discomfort, coughing and anxiety. Loss of appetite, tremors and urinary incontinence also affected some, often linked to medications or comorbidities. Experiencing diverse comorbidities and side effects due to COPD led the participants to feel guilty, recognising their condition as a self-inflicted disease and for being responsible for the health consequences that smoking caused.

When my breathing is erratic, for some reason, I wee. It is when you cannot breathe right you just panic. I know myself, when I’m like that, I have got no control over my bladder. It’s horrible. (…) Unbelievable. You never thought years ago, when you started smoking, all this would happen, but there you go. My own fault. Can’t blame anyone for it.

Participant 15

The use of oxygen affected the participants’ ability to be active and socialise effectively. While it helped with breathlessness, it still restricted their lifestyle, making outings such as going out to the cinema, theatre, or restaurant impossible. Carrying heavy oxygen bottles, as well as the whole process of planning and getting to a place, was reported to be tiring physically and emotionally.

I’ve not set foot inside a theatre for years. First, you have to get from here to the theatre. That takes time. Then, there’s finding your seat and sitting down and watching the play. (…) Also, there’s the weight of either the bottles of oxygen which aren’t light.

Participant 10

Having to plan affected the participants’ social lives as every activity needed to be carefully thought through, thus making it impossible to go out spontaneously. This impacted them emotionally as they felt uncomfortable and worried about spoiling the occasion. Everyday chores took much longer to do than usual, which disrupted having a regular daily pattern.

You get frustrated when you cannot do it. When you feel as if you need to get a bath, you cannot just say ‘right, I’m going to have a bath’. You say: ‘right, will I manage it – all the wee stupid thing you take for granted or you should take for granted’.

Participant 9

In turn, being physically unable to be as active as in the past was related to increased frustration, anxiety or depression.

It’s a frustration, this disease! Not being able to do anything! And that is what I find so frustrating, so upsetting. It’s a fact, but you just can’t do the things you used to do. You need to plan everything. (…) But I’m not depressed. That’s the disease – that’s what it is and you just have to live with that.

Participant 6

Participants pointed to the mind-body connection of experiencing physical symptoms and psychological distress. Anxiety, as well as panic attacks, were common for all participants. Dyspnoea tended to appear abruptly, inducing anxiety or panic attacks. Participants described an association between problems with breathing and panic attacks:

The only symptom is my breathing. When climbing up the stairs, if I get too excited, I panic, and that will result in heavy breathing.

And I think with his breathing now, I think sometimes when his breathing’s bad, he panics, because he maybe thinks something else is going to happen to him, too.

Participant 12 and their wife

The participants reported that anxiety worsened breathlessness, and it was challenging to cope with it and to be able to control it.

Sometimes you just go into your shell — you do not want to talk because if you’re anxious, that’s one of the worst things you can do if you have COPD. Once you get anxious that’s when you’re breathless and properly bring on the attack.

Participant 9

The need for planning was another required adjustment when living with COPD. For example, leaving home was related to asking someone for help, usually relatives, and arriving at the destination earlier to rest if they felt tired. The unpredictability of the disease was a problem for many as it required planning, and it sometimes led to frustration, when it was impossible to adhere to the plan. Leaving home required careful preparation and planning to mitigate anxiety when going out. Factors such as punctuality, fatigue, using stairs and bathroom access were sources of concern.

I’ve got to be early.

…and an hour or two before we go out, to get organised, he gets anxious about going somewhere. We could get to the bottom of the stairs, and he’s got to come back up again.

For the toilet. Aye.

For the bathroom and things like that. He gets anxious just going anywhere that you and I wouldn’t think anything different. He does get anxious.

Participant 21 and their wife

I like to know about the place that I’m going, because it’s always in the back of my mind that there might be a situation where I’m going to, if it’s a lot of stairs, it’s going to be too much for me.

Participant 13

#### Loss of identity

COPD impacted the participants’ ability to move around, be active in their own homes and engage in social and community activities. All participants experienced a sense of loss, as they could not continue undertaking previous social and everyday activities and needed to change their lifestyle because of COPD. The inability to breathe normally forced individuals to become housebound and dependent on others, and they needed to plan whenever they wanted to leave the house. Participants expressed a sense of identity tied to being active and independent. The difficulty laid in the realisation that due to a change in circumstances or health, they were no longer able to maintain the level of activity and independence they once had. The fear stemmed from the prospect of losing this independence, which was a source of anxiety for them.

I was always a person that works and run here and run there. And that’s the hard thing – I can’t do that. (…) I’ve always been very independent and that’s the thing (that) frightens me most. That’s losing the independence I had. That’s what terrifies me.

Participant 6

Being housebound caused a sedentary lifestyle, relinquishing activities like gardening, travel, walks, shopping and home upkeep. Participants described this as being confined and unable to walk long or short distances, missing family parties and relying on relatives and friends to visit them. This led to feelings of loneliness and isolation. Having lost their social life, the participants reflected on the relevance of having social connections.

I don’t have a social life. Only a short time ago I was going out. Now I only go for the appointments. I was out every single day before I got really bad. Now, I don’t go anywhere. Nowhere whatsoever.

Participant 3

Giving up their job and early retirement was common due to suffering physical symptoms and having mobility restrictions. Participants often lost their identity linked to social roles, like being the principal breadwinner or handyman for males, and managing household responsibilities for females. Some participants also complained of losing their role as grandparents as they could no longer take care of their grandchildren or engage in playing with them, attend sporting events and watch them play. Losing their family and job roles led to adverse consequences for their self-confidence. Worsening health conditions resulted in the loss of social roles of the participants, who viewed themselves as useless.

That’s because you look at something when you think a couple of years ago and you could do that in five minutes. But you can’t anymore. That’s what makes you feel redundant - that you need people’s help to do most things that you used to do.

Participant 20

#### Developing lifestyle coping strategies

When living with COPD, most participants developed coping strategies allowing them to participate in limited activities and experience adjustment to the new life. Acceptance was the most common way of coping with the disease. Participants usually accepted the diagnosis, the progressive disease with no cure, and adjusted to the limitations. Understanding the symptoms of the disease and its nature was the first step to acceptance. This helped them to develop or discover new ways in which they could still enjoy their life. When participants acknowledged their restrictions, they were more able to manage their breathlessness attacks. Some participants learnt techniques to calm down and utilised self-management strategies provided by healthcare professionals.

The only way I know is to really get rid of that anxiety because it can become quite frightening. Try to sit down and talk to yourself. That you know what’s happening. You know why it’s happening, and this would be like a kind of self-meditation where you’re the only person who can do that.

Participant 22

Participants were generally satisfied with the knowledge provided by healthcare professionals, which helped them to feel cared for. Being skilfully and empathetically cared for by healthcare workers was highly appreciated by people with COPD, who positively commented on these characteristics of the staff. Healthcare personnel interested in their state, asking ‘how are you?’ and listening to what they had to say were highly esteemed as empathic, understanding and easily approachable medical staff increased patients’ confidence.

I feel supported because there are quite a few people around. More than that, I feel that they are genuinely interested (in me). Not just going through the motions but genuinely interested in how I am doing and that helps me.

Participant 20

Therefore, the quality of support from formal healthcare providers played an essential role in the patients’ perception of being cared for, consequently affecting their well-being. Patients expected healthcare professionals to be knowledgeable about their condition and to know how to manage their disease. Supportive management by professionals also motivated the patients to take care of themselves.

Also, attending support groups, a day hospice, and being surrounded by people in a similar situation, were described as supportive and aided the journey of acceptance. Some participants attempted to avoid overthinking and dwelling on their disease to fight their anxieties. Others tried to make the most of their situation, approaching their anxiety with an acceptance that helped them to avoid pondering. It also meant recognising their own needs and not asking too much of themselves.

I’m not down, as such. I’ve relegated myself to feeling this way, because there’s no point in being happy or sad about it. This is your life, this is the way things are going to be. It’s not going to get any better.

Participant 14

The incurable nature of the disease could yield a feeling of hopelessness about the future. Loss of hope for improvement in life was common and contributed to the feeling of having no purpose in life. Some participants failed to believe that anything could ameliorate their life and mood since it was impossible to improve because it was directly connected with the disease.

I am a great believer it’s not the quantity of your life; it is the quality of your life. And if you do not have quality of life, you do not have anything.

Participant 6

Finding new ways to enjoy their lifestyle, including new hobbies such as plant potting, gardening, pottery or yoga, within their limitations could be another positive consequence of acceptance and adjusting to new life situations. Some tried to make the most of what they had, adapting to the restrictions by using walking aids, a wheelchair or a mobility scooter in order to have some parts of their old life and activities.

Another technique noted was maintaining a normal lifestyle and adopting an attitude of living day by day. For the majority, trying to maintain a normal lifestyle and following the day-by-day strategy was the most crucial in order to cope. Those participants who accepted the diagnosis, coming to terms with the fact that the symptoms would not improve and their lives would be limited, often approached each day with acceptance. Some days seemed better, and some were worse, so it was not easy to predict what the next day would look like. Therefore, due to this unpredictability in terms of experiencing symptoms and mood fluctuation resulting from them, the one-day-at-a-time approach seemed to be the most effective and helpful to some of them.

A difficult day is when you live in bed. You basically can get up, but you’re so breathless and it’s a struggle. (…) I got used to believe to say lately: ‘Today it’s a good day’, but I started again to fear that. So now, when a good day is coming I grab it with my both hands and enjoy them.

Participant 4

For many, holding onto the activities they still could perform was a way to maintain some degree of normality. Those with informal support were more likely to engage in social activities, such as meeting family, friends or attending church, which helped maintain mental sharpness and a sense of purpose.

### Mixed methods findings

Convergence of data was found in the context of experiencing psychological distress. The results from analysing the questionnaires demonstrated that QOL results had a significant relation with psychological distress, concurring with the findings of the interviews, which disclosed that psychological distress was the most influential factor affecting the participants’ well-being. The analysis of the questionnaires indicated that the severity of the disease was significantly correlated with psychological distress and that neither the disease severity nor oxygen use was significantly associated with QOL. Nevertheless, interviews revealed that while oxygen use provided physical relief, such as improved breathing and mobility, it also contributed to social restrictions, including being housebound. The interviews indicated that it was not only the physical symptoms which affected the QOL but also the person’s attitude towards living with COPD, accepting the new lifestyle and trying to adjust to it.

## Discussion

The participants recruited for this study were people with advanced COPD who attended palliative care services, and despite receiving supportive care, they presented high psychological distress measured with HADS. In the present study, anxiety and depression were higher than in most other COPD population studies, with similar age groups and disease severity (Bove et al., 2020; Farver-Vestergaard et al., 2018; Grosbois et al., 2020). Also, our findings showed that increased psychological distress was correlated with increased severity. However, the effect of lung function on the risk of anxiety and depression is not an obvious outcome, as some studies in the past did not report these correlations (Hynninen et al., 2007; Wagena et al., 2005). Moreover, the divergence in results among studies, such as the influence of disease severity on depression but not anxiety in one study (Paine et al., 2019) or the significant impact of severe lung impairment on anxiety and depression in another study (Huang et al., 2021), stresses the heterogeneity of COPD patients’ experiences.

This study reported that higher psychological distress, measured with HADS, was correlated with QOL, measured with MQOL-R. It is a relatively recent questionnaire published in 2017 (Cohen et al., 2017), and as a consequence, there are few published papers to compare with. So far, this has been the first study which employed MQOL-R to investigate the COPD population. Until now, only Althaus et al. (2018) had conducted a study using MQOL-R with palliative care patients. Comparing their findings with those from the present study, it is evident that people with COPD experience poorer QOL and more pronounced anxiety and depression than those with cancer or other diagnoses in palliative care. Studies exploring the COPD population which used different instruments, found a corresponding tendency. Namely, a higher QOL positively correlates with psychological well-being (Jang et al., 2019) and physical restrictions substantially limit HRQOL (Brandl et al., 2018; Hynninen et al., 2007). Consequently, the collective evidence underscores the significance of psychological distress in influencing the QOL of individuals with COPD, as consistently observed across various studies and instruments.

People with advanced COPD experience a high physical symptom burden, which leads to a loss of functionality and a high level of psychological distress. Thus, person-centred care is recognised as potentially beneficial for these patients in addition to acknowledging their social needs (Gardener et al., 2018). British and Scottish strategy documents emphasise the need to stress these patients’ physical, psychological, social and spiritual needs through a holistic, supportive and person-centred model of care (National Palliative and End of Life Care Partnership, 2021; Scottish Government, 2019). Furthermore, person-centred care is related to the biopsychosocial model approach and can improve patient outcomes (Smith et al., 2013; Turabian, 2018; van Dulmen et al., 2015; Weiner et al., 2013).

This study showed that the principles of the biopsychosocial model in the management of long-term conditions can enhance patient care by promoting an integrated and holistic approach that recognises the interplay of biological, psychological, and social aspects of the disease. Current guidelines have acknowledged the biopsychosocial impact of living with a chronic condition and suggested that people with chronic diseases, such as COPD, should receive integrated care programmes which are patient-centred rather than solely disease-centred (Scottish Government, 2017; van Dulmen et al., 2015). Ultimately, this approach aims to improve multidisciplinary collaboration and physician-patient relationships while empowering patients to participate more actively in their disease management (Kusnanto et al., 2018). Previous research has also stressed that attention should be paid to using shared decision-making and connecting to the goals and motivation of the patient rather than only providing information and advice, which may fail to contribute to lifestyle changes (Elwyn et al., 2012; Marteau et al., 2015). Nonetheless, Hillebregt et al. (2017) showed that patients with COPD and healthcare professionals had problems identifying patient-orientated goals and expectations. A possible explanation for these findings can be that patients were not used to having a proactive role due to being framed in a traditional and medically oriented structure of the consultation.

For years, the person-centred approach and empathy have been proposed as a shared standard for healthcare workers (Kerasidou and Horn, 2016; Santana et al., 2018; Suazo et al., 2020). Additionally, this study showed that being understood by healthcare professionals and having a complete clarification of the diagnosis and its progress gave participants of this study a feeling of reassurance. It was of principal value to participants to be treated as a person with deference and empathy. Similarly, people with refractory breathlessness presented results comparable to this study, reporting that participants considered healthcare personnel to be specialists in overseeing their disease in person-centred care, and particularly valued being approached with respect to their dignity (Gysels et al., 2016). Therefore, in terms of the biopsychosocial model, recognising patients’ emotional states can facilitate healthcare providers in the effort to give individualised and holistic care.

### Strengths and limitations

This research brought together patients’ experiences and views and then supplemented their voices with an objective measurement of psychological distress and QOL from validated questionnaires. It was demonstrated that using both methods can accomplish a desired aim by employing an embedded design. In this study participants were recruited from four services, allowing us to capture a wide range of experiences and needs of people with COPD across various palliative care environments. Although the sample size was sufficient for this mixed methods concurrent study, a more diverse sample, including participants from other services or regions, could be informative. Additionally, the use of purposive sampling inherently limits the representativeness of the sample and may have introduced selection bias and limited the generalisability of the findings. Therefore, the results may not represent the full spectrum of patients, particularly those who are not referred to services or have not been doctor-diagnosed.

### Future directions

These findings prompt further exploration into the interplay of various factors, including socioeconomic factors, such as social deprivation, and comorbidities which have not been explored in this study. Given the limited representation of people with COPD in day hospices, conducting a nationwide study is advised to allow for further quantitative analysis of the factors affecting the psychological burden.

## Conclusion

This study demonstrated clear associations between biological, psychological and social aspects of living with advanced COPD. Experiencing physical symptoms affects everyday life, as it limits daily activities and the individual’s independence, while it increases dependence on others, contributing to developing frustration, dismay and psychological distress. Social aspects of life can also be affected due to patients’ being housebound, feeling isolated and unable to participate in previously attended social activities. Finally, these losses can force patients to develop coping and adapting strategies. The principles of the biopsychosocial model should be considered in palliative care for patients with COPD to promote positive outcomes for patients by targeting the self-management of physical symptoms, addressing social needs and reducing psychological distress.
